# Effects of Valproic Acid Embryonic Exposure on Zebrafish: A Systematic Review and Meta-Analysis

**DOI:** 10.3390/neurosci5040046

**Published:** 2024-12-07

**Authors:** Bernardo Flores-Prieto, Jorge Manzo-Denes, María Elena Hernández-Aguilar, Genaro Alfonso Coria-Avila, Deissy Herrera-Covarrubias, Gonzalo Emiliano Aranda-Abreu, Fausto Rojas-Durán, César Antonio Pérez-Estudillo, Jorge Suárez-Medellín, María Rebeca Toledo-Cárdenas

**Affiliations:** Instituto de Investigaciones Cerebrales, Universidad Veracruzana, Xalapa 91070, Mexico; zs200226664@estudiantes.uv.mx (B.F.-P.);

**Keywords:** neurodevelopmental disorders, zebrafish, systematic review, meta-analysis, autism spectrum disorder

## Abstract

Exposure to valproic acid (VPA) during embryogenesis has become a valuable tool for modeling neurodevelopmental disorders in animal models such as zebrafish (*Danio rerio*). This article examines the effects of embryonic exposure to VPA in zebrafish on the basis of 39 articles sourced from PubMed and Google Scholar. We conducted a systematic review and meta-analysis to elucidate the common impacts of VPA exposure and reported that VPA significantly altered development at various levels. Behaviorally, zebrafish exposed to VPA exhibit notable changes in their social interaction patterns. Physiologically, VPA exposure leads to significant alterations, including decreased heart rates, increased mortality rates, and pronounced morphological abnormalities. Pharmacological exposure has been linked to neuroanatomical and neurochemical changes. At the genetic level, VPA exposure is associated with the differential expression of genes involved in neurodevelopment and neuronal function. The synthesized data from these studies underscore the utility of zebrafish as a model organism for investigating the effects of teratogen exposure on neurodevelopment.

## 1. Introduction

Valproic acid (2-propylpentanoic acid) is an antiepileptic drug and mood stabilizer that has been widely used since 1974. Its therapeutic action increases the level of the neurotransmitter GABA (gamma-aminobutyric acid) in the central nervous system (CNS), significantly reducing the occurrence of epileptic seizures and modulating neuronal excitability [1,2]. Additionally, VPA acts as a potent epigenetic modulator through its inhibition of histone deacetylases. However, prenatal exposure to VPA is associated with major congenital malformations and neurodevelopmental disorders, including attention deficit hyperactivity disorder and autism spectrum disorder (ASD) [3].

Notably, exposure to VPA during the first trimester of gestation in humans is associated with up to a 10% increase in the likelihood of meeting the diagnostic criteria for ASD [4,5], which are broadly classified into three key areas: (1) social deficits, (2) communication deficits, and (3) cognitive and behavioral inflexibility. ASD is frequently accompanied by a wide range of comorbidities, including sensory disorders, intellectual disability, anxiety, depression, cardiovascular conditions, and epilepsy, among others [6,7]. In the United States, epidemiological studies report a prevalence of ASD of approximately 1 in 40 individuals. Although no global statistical assessment exists, it is estimated that the prevalence is similar worldwide [8]. Regarding the potential underlying causes of ASD, both experimental and clinical evidence suggest a combination of genetic, epigenetic, and environmental factors that influence its development [9,10]. Therefore, understanding the origins, prognoses, and potential treatments that can improve the quality of life of individuals with ASD is highly important.

For this reason, in the field of biomedical and experimental research, animal models that allow us to study ASD and its possible treatments are needed so that the information obtained allows us to establish translational correspondences applicable to humans. Currently, multiple animal models of different species reproduce the characteristics of ASD and allow us to explore their neurobiology and genetics [11,12]. One of these models involves zebrafish exposed to VPA, in which behavioral alterations such as social deficits, cognitive deficits, behavioral rigidity, and locomotor alterations consistent with an ASD model have been reproduced, as previously reviewed by our research group [13].

Compared with other models, the zebrafish model treated with VPA offers several advantages, including rapid development, embryonic susceptibility to teratogens, transparency during embryonic stages, and a high reproductive rate. Consequently, its use as a model for studying ASD at the behavioral, cellular, molecular, and genetic levels has become increasingly popular. However, methodological discrepancies currently complicate the comparative analysis of results across multiple studies [11]. Therefore, it is important to analyze the common effects of embryonic exposure to VPA in the context of its use as a biological model for the study of developmental disorders.

In this review, we focus on analyzing the effects of early VPA exposure in zebrafish, with a particular emphasis on neurobiological and genetic aspects. Key methodological factors, including the timing of pharmacological exposure and the concentration of VPA used, were also considered.

## 2. Materials and Methods

### 2.1. Research, Identification, and Inclusion Criteria

**Figure 1:**
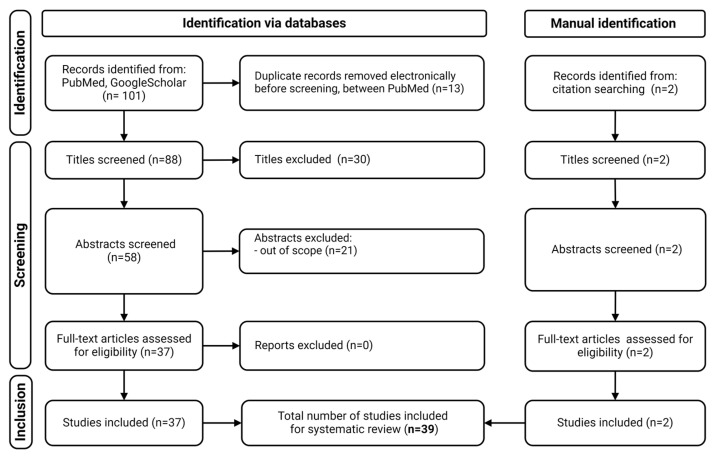
PRISMA flowchart of the review process.

We conducted a systematic search in two databases (PubMed and Google Scholar) via the terms “zebrafish” and “valproic acid” or “valproate”. The search was restricted to original articles published between 2014 and 2024. A total of 101 articles were identified, and 2 additional articles were found manually. Review articles, duplicate entries, and studies involving posthatch exposure were excluded. The titles and abstracts of all the articles were reviewed, and only those in which VPA exposure occurred during the first 72 hpf (hours postfertilization) were selected. Low-quality studies lacking statistical analyses or essential methodological details were excluded. After a filtering process on the basis of relevance and data quality, a total of 39 articles were included in the final analysis (Figure 1). In all these studies, zebrafish were exposed to VPA during critical periods of embryonic development, and behavioral, morphological, molecular, or genetic changes were evaluated. The protocol was registered on OSF (https://osf.io/frtgq, accessed on 1 November 2024).

Our systematic analysis focused on the biological effects of embryonic VPA exposure, as the behavioral effects have been thoroughly reviewed in a previous study [13]. For the meta-analysis, we included articles that provided complete and high-quality statistical data, allowing for a robust comparative analysis. The selected studies met the following criteria: (1) original research conducted with zebrafish exposed to VPA, (2) pharmacological exposure via immersion during the prehatching period, and (3) results reported as the mean and standard deviation (MD ± SD) or mean and standard error (MD ± SE).

### 2.2. Data Treatment

All the articles included in this review were organized into a spreadsheet (Microsoft Excel, Microsoft 365 MSO version 2410), and key data, including the year of publication, title, authors, journal, VPA concentration, start and end of pharmacological exposure, effects at various levels, conclusions, statistical data, and specific notes about each article, were extracted. When applicable, quantitative units were standardized to a common metric to facilitate statistical analysis. In some cases, statistical data were extracted from published graphs via the WebPlotDigitizer tool (version 4.7) [14]. The extracted data included sample size, standard deviation, and mean ± standard error. Studies that used different concentrations of VPA or varied exposure periods but compared them against the same control group were treated as independent experimental units.

### 2.3. Data Analysis

For the meta-analysis, we used the RStudio meta package [15] to calculate the standardized mean difference (SMD) as an estimate of the effect size across two types of outcomes: continuous and binary data. Shoaling behavior and social preference, gene expression, and heart rate were considered continuous data, whereas mortality was considered binary data. For continuous outcomes, MDS was estimated via Hedges’ g statistic, and interstudy variance (τ^2^) was calculated via the DerSimonian–Laird method [15,16,17]. The effect size was considered significant with a 95% confidence interval (CI). Statistical heterogeneity was calculated via Higgins I^2^ [15,18]. Subgroup tests were performed according to the beginning of the period of exposure to VPA. Similarly, meta-regressions were performed according to the concentration of VPA with the “metareg” function. The results were considered significant at *p* < 0.05. All procedures involving continuous data were performed with R’s “metacont” function [15].

For binary data, we calculated both fixed-effect and random-effect estimates for the meta-analysis using R’s “metabin” function. Specifically, the Mantel–Haenszel (MH) method was applied, along with the Hedges estimator for interstudy variance (τ^2^), to calculate the risk ratio (RR) with a 95% CI [15,19]. Residual heterogeneity was assessed using Higgins’ I^2^ statistic [15,18]. Subgroup analyses and meta-regressions were conducted in the same manner as for the continuous data.

### 2.4. On Methodological Aspects

One of the main obstacles to comparing results across different protocols is methodological variability. To address this, all the articles included in this review shared two key aspects: (a) VPA exposure was conducted via the incubation medium, and (b) pharmacological exposure occurred during the embryonic period. Despite multiple methodological differences among the studies, we focused on two relevant variables: (a) the concentration of VPA administered and (b) the timing of VPA exposure. For the embryonic exposure stage, we referred to the developmental stages described by the Zebrafish Information Network (ZFIN) (from Zfin.org website: https://zfin.org/zf_info/zfbook/stages/ (accessed on 22 January 2024)).

## 3. Results

### 3.1. Systematic Review

A total of 101 articles published between 2014 and 2024 were obtained from a systematic search of PubMed and Google Scholar. After removing duplicate articles (n = 13) and excluding those that did not meet the inclusion criteria (n = 49), one additional article was manually included. In total, 39 original research articles published over the past 10 years were selected for this review, all of which involved zebrafish embryos exposed to VPA.

The studies initiated VPA exposure at different stages of embryonic development: 10 during the zygote stage (0–0.75 hpf), 1 during the cleavage stage (0.75–2.25 hpf), 7 during the blastula stage (2.25–5.25 hpf), 10 during the gastrula stage (5.25–10.33 hpf), 5 during the segmentation stage (10.33–24 hpf), and 3 during the pharyngula stage (24–48 hpf). However, the duration of exposure varied considerably across the protocols. VPA concentrations ranged from 1 μM to 2560 μM, with all dosing protocols carried out by immersion in the medium. Qualitative analysis of the overall reported results indicated significant developmental alterations due to pharmacological exposure, regardless of the exposure period. However, certain periods of increased susceptibility to pharmacological insults have also been identified.

#### 3.1.1. Embryonic Exposure to VPA Disrupts Normal Development in Zebrafish

Five studies specifically examined mortality and reported that exposure to VPA at concentrations ranging from 0.1 to 693.43 μM increased mortality in a concentration-dependent manner. Furthermore, the data suggest that earlier and more prolonged pharmacological exposure increases susceptibility to mortality [20,21,22,23,24]. Similarly, five studies investigating cardiac activity following embryonic exposure to VPA reported significant functional and anatomical changes in the cardiovascular system [22,24,25,26,27]. Key alterations include changes in heart morphology [26] and pericardial edema [20,21,23,28,29,30,31], as well as an increase in the size of the pericardial sac [22,26,27,32,33]. One of the most consistent reported findings was the alteration in heart rhythm, which is consistent with reports of various heart malformations [22,24,25,26,27]. Additional findings include an increase in the number of apoptotic cardiac cells [26], disruptions in cardiomyocyte formation and migration, and delayed heart development [33].

Moreover, 12 studies evaluated bone system morphology and development, revealing VPA-induced changes within a concentration range of 1–1500 μM. These alterations include changes in craniofacial morphometry and cartilage structures [29,31,34,35,36], spinal curvature [21,23], microcephaly [28], or macrocephaly [20].

However, no dose-dependent correlations or specific periods of drug exposure have been consistently reported. On the other hand, 5 studies identified alterations at the level of the CNS at concentrations ranging from 5 to 1000 μM. Among the reported changes were deformations in the preoptic area, thalamus, and hypothalamus; enlargement of the cerebellum and ventral thalamus; and a reduction in the volume of the interpeduncular nucleus and anterior commissure [37]. Severe alterations in the general brain structure were observed starting at 60 μM, with dose-dependent effects at concentrations of 150, 320, and 730 μM. More pronounced alterations were linked to prolonged exposure periods (0–24 hpf, 0–48 hpf, 0–72 hpf, or 0–96 hpf) [30], including delayed optic tectum development and a reduction in neuropil volume [38]. While midbrain size decreases, there is notable widening of the midline gap in the hindbrain, as well as concentration-dependent perturbations in secondary motor neuron neurite outgrowth (0.1 μM, 0.3 μM, 1 μM, 3 μM, 10 μM, and 30 μM) [28]. At concentrations of 0.1μM, and 0.3μM, axonal defects, such as excessive branching, innervation of neighboring myotomes, and ectopic branching, were observed. At 10 μM, embryos presented missing axons or excess axonal branching [28] and decreased molecular layer volume in the cresta cerebelli of the medial cerebellum [39].

In summary, all the studies reviewed indicate significant disruptions in the health and neurodevelopment of zebrafish exposed to VPA, which is consistent with previous reports highlighting its potent teratogenic effects.

#### 3.1.2. Embryonic Exposure to VPA Triggers Molecular and Epigenetic Changes

The role of VPA as a modulator of epigenetic processes has been extensively studied in zebrafish through embryonic administration protocols. Pharmacological concentrations range from 5 μM to 2000 μM, with exposure periods spanning the entirety of zebrafish embryogenesis. In this review, 31 studies were analyzed, revealing significant molecular and genetic alterations in response to VPA exposure. These include disruptions in neuronal proliferation and differentiation, as well as modifications in neurotransmission systems.

In this sense, an increase in the number of BrdU-positive cells in the hindbrain has been reported, but a decrease in the percentage of differentiated cells in neurons, in both cases, is dependent on an increase in the concentration of VPA (1000 μM, 1500 μM, and 2000 μM) [40]. Interestingly, exposure to 5 μM resulted in increased *HH3* expression, suggesting increased cell proliferation, as well as increased immunolabeling for HuC/D+NEUN+ and PCNA+, indicating an increase in the proportion of mature neurons [20]. In contrast, in situ hybridization techniques revealed a decrease in cellularity in the preoptic area and optic tectum, accompanied by an increase in the number of apoptotic cells in a dose-dependent manner at concentrations of 150, 320, and 730 μM [30].

At the neurochemical level, glutamatergic expression is significantly affected by exposure to VPA at a concentration of 50 μM. An increase in glutamatergic activity was observed in regions such as the optic tectum, epithelium, and olfactory bulb, whereas a decrease was noted in the subpallium, hypothalamus, thalamus, and interpeduncular nucleus [37]. An increase in glutamatergic activity was observed in regions such as the optic tectum, epithelium, and olfactory bulb, whereas a decrease was noted in the subpallium, hypothalamus, thalamus, interpeduncular nucleus [41], and histamine [42]. Similarly, VPA at concentrations of 30 and 40 μM increases acetylcholinesterase activity in the brains of adult fish exposed during embryogenesis [21]. Furthermore, oxytocin receptor expression is reduced following pharmacological exposure to VPA [43].

With respect to the epigenetic changes induced by VPA, significant alterations in genes involved in proliferation, differentiation, neuromodulation, neuroplasticity, and the formation and function of the cardiovascular system have been reported. Specifically, epigenetic dysregulation markers, such as increased and accumulated histone H3 and H4 in a dose-dependent manner (50, 100, and 150 μM), were identified [44]. Additionally, VPA exposure led to a dose-dependent increase in the expression of *mbd5*, a gene involved in chromatin regulation, at concentrations of 15 and 50 μM [45]. Other genes, such as *KAT7b*, *HAT1*, *MEAF6*, *HDAC1*, and *HDAC3*, also exhibited dose-dependent increases at concentrations of 5.55 μM, 27.74 μM, 138.69 μM, and 693.43 μM, respectively. In contrast, some genes associated with DNA methylation presented mixed responses. For example, *dnmt1* expression decreased at 5.55 μM, whereas other methyltransferase genes, such as *dnmt3*, *dnmt4*, *dnmt5*, *dnmt6*, *dnmt7*, and *dnmt8*, increased in a dose-dependent manner at 5.55 and 27.74 μM [22].

In terms of synaptic function, VPA exposure altered the expression of several related genes, including *Shank3a*, *Shank3b*, *Shank2a*, *Shank3b-1*, *Shank3a-1*, *Shank3a-4*, *Shank3b2*, and *Shank3a3* [43,45,46,47]. Similarly, decreased transcription of *Akt* (*pAkt*/*Akt*) and *mTOR* (*pmTOR*/*mTOR*) has been reported in the brain tissue of adult zebrafish treated with VPA at 30 and 40 μM [22]. VPA also triggered an increase in the expression of genes involved in the *Wnt* signaling pathway, such as *cdh8*, *ctnnb*, *gsk3 beta*, and *lrp6*, which are critical for proper CNS development [48,49]. Moreover, decreased expression of proneural genes, including *ascl1a* and *ascl1b*, was observed following low pharmacological exposure to VPA at concentrations of 1, 2.5, and 5 μM [50], along with reduced neural proliferation marked by tsclb in a dose-dependent manner at 25 and 50 μM [45]. Similarly, VPA increased the expression of *dlx2* [35] *mbd5*, and *fgf* [31,48], which are involved in neuronal differentiation. The expression of several morphogens crucial for development, Such as *sox2* [41], *nkx3.2,* and *fgf3* [35,48], is also increased by exposure to VPA. The expression of genes related to cell and receptor adhesion, such as *NRXN1* and *NGLN3*, decreases after exposure to 75 μM VPA [45,46]. Additionally, genes associated with the formation and maintenance of the cytoskeleton, including *actn4*, *gria3*, and *grid1*, were also downregulated [45].

Similarly, the retinoic acid regulatory system, which plays a key role in the formation and early differentiation of the CNS, was also affected by VPA exposure. Specifically, increased *Aldh1a2* and *cyp26a1* expression was observed in the cerebellum, motor spinal cord, and optic tectum in a dose-dependent manner at 150 μM *Aldh1a2* [29,30].

Similarly, genes associated with neurotransmission systems also exhibited significant alterations following embryonic exposure to VPA. For example, genes linked to the glutamatergic system, such as *grm5a*, presented increased expression at 5 μM [51] but decreased expression when exposed to 75 μM [46,48]. Additionally, VPA exposure reduces the expression of genes involved in the serotonergic system, including *5htr3a*, *5htr3b*, and *tph2* [50], as well as the dopaminergic system, such as *dbh* [42,48,50]. Similarly, the expression of genes related to the histaminergic system, such as *hdc*, *hrh1*, *hrh2*, and *hrh3*, decreases [42]. The purinergic system was also affected, as suggested by the increase in the transcription rate of *adsl* [31,45].

Moreover, significant changes in genes related to the formation and function of the cardiovascular system, such as *myl7* [25], *col9a1b*, *sp7*, and *axin2* [35], have been detected in response to pharmacological exposure. In addition, the relative expression of *tbx5b* decreases with increasing relative expression of *tbx5a* [31].

### 3.2. Meta-Analysis Results

#### 3.2.1. Embryonic Exposure to VPA Increases the Mortality Rate of Zebrafish

**Figure 2:**
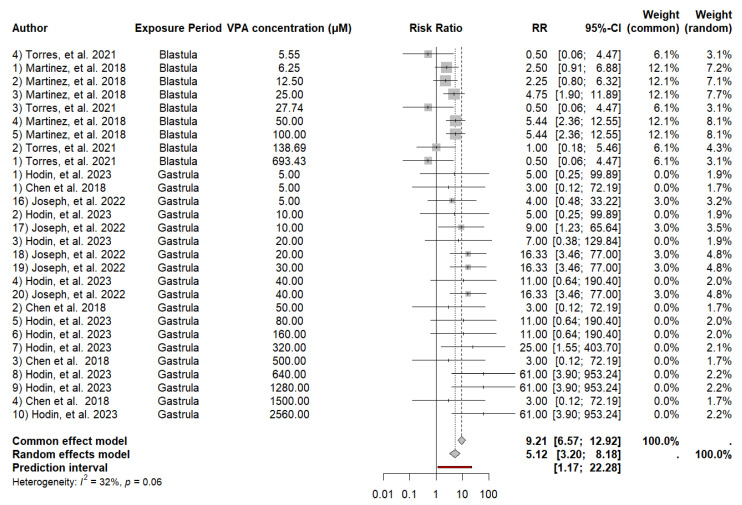
Forest plot of the RR and 95% CI for the effect of the VPA concentration on mortality in zebrafish.

We observed that embryos exposed to valproic acid presented increased mortality at the different concentrations evaluated. Common effects analysis revealed an RR of 9.2121 (*p* < 0.0001), and random effects analysis revealed an RR of 5.1158 (*p* < 0.0001), indicating a significant effect of VPA on zebrafish mortality. Heterogeneity between studies was moderate (I^2^ = 31.8%, τ^2^ = 0.4602, *p* = 0.0561). Meta-regression analysis revealed a significant relationship between mortality and the period of initiation of exposure to VPA (SE = 0.33, *p* = 0.0004), indicating that exposure during gastrulation is associated with increased mortality compared with exposure during blastulation. We performed a meta-regression to explore the effect of the VPA concentration on mortality. A positive trend was observed, with an estimate of 0.0009, but the test did not reach statistical significance (*p* = 0.0918). This finding indicates that although there is a trend toward a greater effect with higher concentrations of VPA, the evidence is not strong enough to conclude a significant relationship in this study (Figure 2).

#### 3.2.2. Embryonic Exposure to VPA Alters Heart Function in Zebrafish

**Figure 3:**
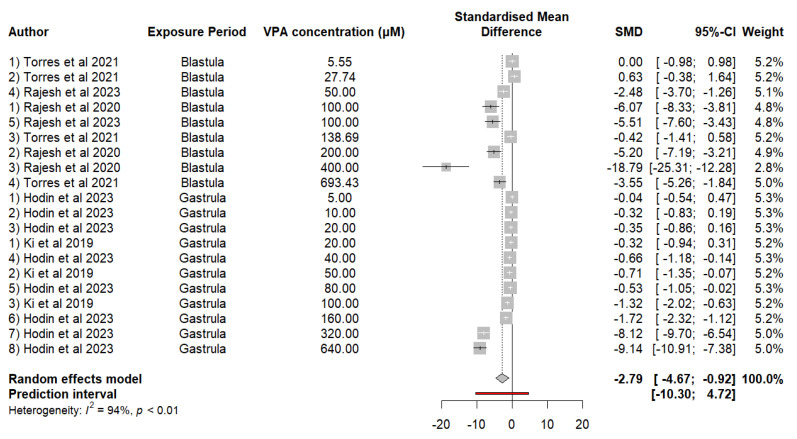
The forest plot shows the SMD and 95% CIs for the effects of the VPA concentration on heart rate in zebrafish.

Our findings on the heart rate of embryos exposed to VPA indicate a significant decrease compared with that of controls. Analysis of the data revealed an SMD of −2.7927 (*p* = 0.0057), indicating a noticeable decrease in heart rate due to exposure to VPA. The analysis of heterogeneity revealed a high degree of variability between studies (I = 93.9%, *p* < 0.0001), so differences in results cannot be fully explained by chance. The difference in effect between exposure periods was not statistically significant (*p* = 0.3196), suggesting that the impact of VPA on heart rhythm does not vary significantly between the blastula and gastrula. The estimate for τ^2^ is 12.1363, and that for τ is 3.4837, underscoring the presence of considerable heterogeneity in the effects reported between studies. Meta-regression was used to evaluate the effect of the VPA concentration on heart rate. The results revealed a significant negative relationship between the VPA concentration and heart rate, with an estimate of −0.0118 (SE = 0.0034, t = −3.4997, df = 18, *p* = 0.0026). This finding suggests that at higher concentrations of VPA, the heart rate tends to decrease to a greater extent (Figure 3).

#### 3.2.3. Embryonic Exposure to VPA Decreases Social Affiliation in Zebrafish

**Figure 4:**
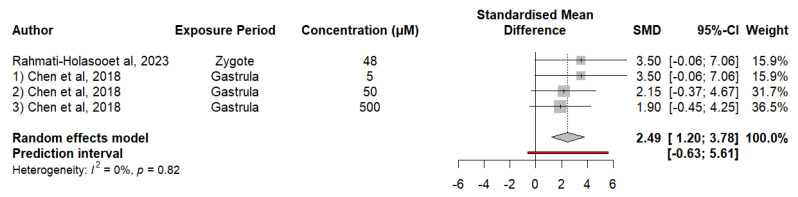
The forest plot shows the SMD and 95% CI for the effect of the VPA concentration on shoaling behavior in zebrafish.

Our results on shoaling behavior in zebrafish exposed to VPA indicate a significant difference in affiliation behavior between groups exposed to VPA. The SMD effect for shoaling behavior was 2.4892, which indicates a significant effect of VPA on shoaling behavior; in this case, it could cause a greater distance between individuals, which may indicate an alteration in the normal social behavior of zebrafish. The I^2^ value was 0%, with a *p*-value of 0.82, confirming that heterogeneity between studies was not significant. We performed a meta-regression; however, the coefficient for the VPA concentration was −0.0021, indicating that although there was a trend toward a decrease in shoaling behavior with increasing VPA concentration, this effect was not statistically significant (*p* = 0.3308). These findings suggest that the VPA concentration does not significantly explain the variability in shoaling behavior in the studies reviewed (Figure 4).

#### 3.2.4. Embryonic Exposure to VPA Alters Social Preference in Zebrafish

**Figure 5:**
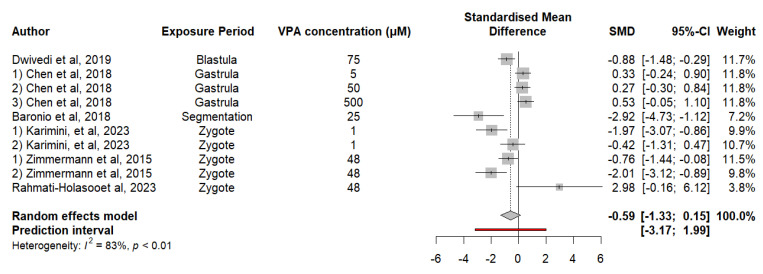
The forest plot shows the SMD and 95% CI for the effect of the VPA concentration on social preference in zebrafish.

The results of the analysis suggest that there is a tendency toward social deficit in fish exposed to VPA, but this trend is not significant. Analysis of the data revealed a difference in the MDS of −0.5897 (*p* = 0.1970), suggesting a decrease in social preference due to exposure to VPA, although this effect was not statistically significant. The heterogeneity of the results obtained between the studies, with an I^2^ of 82.7% (*p* < 0.0001), indicates high variability, which partially explains the lack of statistical significance. The estimate of τ^2^ is 1.1086, and τ is 1.0529, emphasizing such heterogeneity in the reported effects. The results of the meta-regression revealed that although the concentration of VPA had an estimated positive effect of 0.0027, this effect was not statistically significant (*p* = 0.3523). The 95% confidence interval for the coefficient of the VPA concentration is [−0.0036; 0.0091], suggesting that the effect of concentration on social preference may be very small or nonexistent. The moderator test did not reveal a significant difference (F (df1 = 1, df2 = 8) = 0.9753, *p* = 0.3523), reinforcing the idea that the VPA concentration did not have a significant effect on social preference in the included studies (Figure 5).

#### 3.2.5. Embryonic Exposure to VPA Alters the Gene Expression of *Shank3a* in Zebrafish

**Figure 6:**
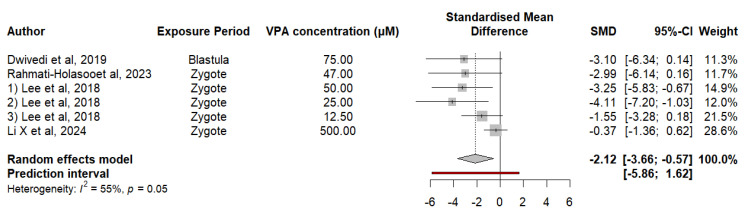
The forest plot shows the SMD and 95% CI for the effect of VPA on *Shank3a* expression in zebrafish.

Studies analyzing the expression of the *Shank3a* gene in zebrafish exposed to VPA acid have revealed a significant decrease compared with that in controls; in this sense, the data revealed an SMD difference of −2.1188 (*p* = 0.0169), which suggests a notable decrease due to exposure to VPA. Heterogeneity analysis revealed a moderate degree of variability between studies (I^2^ = 55.1%, *p* = 0.0487), indicating that differences in outcomes cannot be fully explained by chance. The estimate for τ^2^ is 1.3572, and that for τ is 1.1650, indicating considerable heterogeneity in the effects reported between studies. Meta-regression was used to evaluate the effect of the VPA concentration on *Shank3a* expression. The results revealed a significant positive relationship, with a regression coefficient of 0.0048 (SE = 0.0016, t = 2.9621, df = 4, *p* = 0.0415). These findings suggest that at relatively high concentrations of VPA, *Shank3a* expression tends to increase. The mixed-effects model explained 84.98% of the total heterogeneity, suggesting that the VPA concentration is an important moderator of the observed variability. The residual heterogeneity, measured by τ^2^, was 0.2039 (SE = 1.3762), indicating that the amount of variability was not explained by the VPA concentration (Figure 6).

#### 3.2.6. Embryonic Exposure to VPA Alters the Expression of the *adsl* Gene in Zebrafish

**Figure 7:**
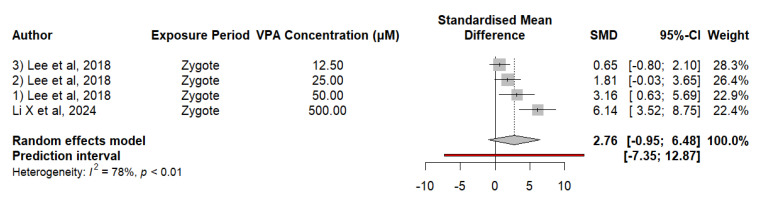
The forest plot indicates the SMD and 95% CI for the effect of VPA *adsl* expression in zebrafish.

The findings of *adsl* expression in zebrafish exposed to VPA indicate an upward trend compared to controls, without reaching significance. The analysis of the data revealed an SMD of 2.7607, suggesting an increase in *adsl* expression due to exposure to VPA, although the result was not statistically significant (*p* = 0.0990). The analysis of heterogeneity revealed a high degree of variability between studies (I^2^ = 78.1%, *p* = 0.0033), indicating that differences in results vary greatly between studies. The estimate of τ^2^ is 4.1822, and τ is 2.0450, which in turn underscores the presence of considerable heterogeneity in the reported effects between trials. Meta-regression was used to evaluate the impact of the VPA concentration on *adsl* gene expression and revealed a significant positive relationship between the VPA concentration and *adsl* gene expression, with a regression coefficient of 0.0101 (SE = 0.0031, t = 3.2197, df = 2, *p* = 0.0844). These results suggest that at relatively high concentrations of VPA, the expression of the *adsl* gene tends to increase. The mixed-effects model explained 97.17% of the total heterogeneity, indicating that the VPA concentration is an important moderator of the variability observed in these studies. The residual heterogeneity, measured by τ^2^, was 0.1183 (SE = 1.0241), indicating that a small amount of variability remains unexplained by the concentration of VPA (Figure 7).

#### 3.2.7. Embryonic Exposure to VPA Alters the Expression of the *mbd5* Gene in Zebrafish

**Figure 8:**
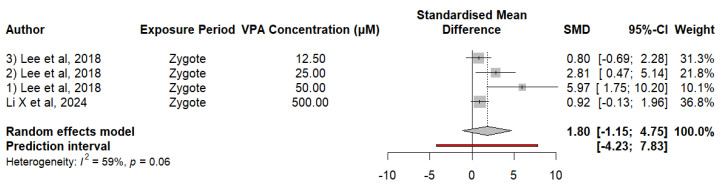
The forest plot shows the SMD and 95% CI for the effect of VPA on *mbd5* expression in zebrafish.

The expression of the *mbd5* gene in zebrafish exposed to VPA does not indicate a significant difference compared to controls. Analysis of the data shows an MDS of 1.8009 (*p* = 0.1472), suggesting that exposure to VPA has no noticeable effect on *mbd5* gene expression. Heterogeneity analysis revealed a moderate degree of variability between studies (I^2^ = 58.6%, *p* = 0.0645). The estimate of τ^2^ is 1.3586, and that of τ is 1.1656. This indicates considerable heterogeneity in the effects reported between studies. Meta-regression evaluated the effect of VPA concentration on *mbd5* expression, and the regression coefficient for the VPA concentration was −0.0032 (SE = 0.0054, t = −0.6026, df = 2, *p* = 0.6080). These findings suggest that the VPA concentration does not significantly affect gene expression. The mixed-effects model accounted for 0.00% of the total heterogeneity, indicating that VPA concentration is not an important moderator for the variability observed in studies on *mbd5* expression. The residual heterogeneity, measured by τ^2^, was 3.8238 (SE = 5.6967), indicating that a considerable amount of variability remains unexplained by the concentration of VPA, as shown in Figure 8.

## 4. Discussion

### 4.1. General Methodological Aspects

Embryonic exposure to drugs with teratogenic effects is a widely used methodology for research into neurodevelopmental disorders [52,53,54]. The first studies that linked exposure to VPA with neurodevelopmental alterations arose from clinical studies derived from the side effects of the drug as an antiepileptic [55]. In this sense, the reports of infants with diagnostic criteria consistent with ASD were linked to in utero exposure to VPA and were used as a basis for its use as an inducer of neurodevelopmental alterations in animal models [1]. In principle, murine models such as rats or mice are generated through intraperitoneal injections during the first weeks of gestation. In these species, VPA dosing had results similar to those observed in humans, i.e., phenotypes characteristic of neurodevelopmental disorders. In general, reports have focused on the behavioral manifestations of the social, cognitive, and locomotor domains [52]. These models have also provided highly relevant information for understanding the underlying neurological manifestations of these behavioral deficits. These include excitation/inhibition imbalance, synaptic alterations, neuroinflammation, alterations in neurogenesis, and epigenetic changes [1].

The growing interest in genetic aspects underlying neurodevelopmental disorders has led to alternatives in biological models to study this field. Zebrafish is one of the species that has aroused great interest in this regard because of its multiple methodological, technical, and economic advantages for use both in the field of neurosciences and in biomedical sciences in general, where it has become popular and consolidated [11,56]. This is why strengthening the methodological congruence between the vastness of approaches reported has become a technical and theoretical necessity. We propose that the first method to this problem can be from a qualitative analysis perspective through a systematic and quantitative review through statistical approximations, which allows the identification of the main coincidences between the results obtained from different studies. Knowing the general panorama of the information of this model can be useful for making decisions of experimental relevance, such as the period of exposure and the pharmacological concentration used.

From the general analysis of the works synthesized here, it is important to highlight the high degree of methodological heterogeneity. This methodological variability makes it difficult to analyze the results together, mainly because of the variability in the concentration of dosed VPA, the type of analysis used, the period of onset and end of exposure, the age of assessment, and the technique used for it. However, a measured and meticulous comparison of the experiments, techniques, and results reported allowed common and congruent effects to be extracted; however, discrepancies or contrary results between different studies also allow the identification of areas of experimental opportunity that need clarification and further investigation.

### 4.2. Alterations in General Development

Taken together, these results emphasize the teratogenic potential of VPA during embryonic exposure. VPA compromises early development, because of its ability to regulate the HDAC (Histone deacetylase) system. The dysregulation of this system leads to significant epigenetic changes that alter the orchestrated expression of genes essential for proper development, mainly of the nervous system [57]. In zebrafish, the drug has been shown to be detectable in the embryo from the first 24 h of exposure [58]. In all the studies considered here, the drug was exposed for more than 24 h, and the solution was replaced to ensure its pharmacological action. Therefore, we believe that the action of VPA on the HDAC system partially explains the reported effects.

The increase in the mortality rate, which is dependent on the period of exposure and tends to increase in dose-dependent mortality, suggests the existence of failures in the formation of systems indispensable for survival, such as the cardiovascular system and CNS. Specifically, during the blastula period (2–5 hpf), the first cell divisions begin, which are orchestrated by morphogenes and transcription factors that shape the original embryonic layers. Early exposure to VPA significantly alters the expression of multiple genes involved in cell differentiation, proliferation, and maintenance processes, which partially explains the alterations in the CNS and cardiovascular structures.

It is crucial to recognize that the effects of VPA are stage-specific during embryonic development. During the blastula stage (2–5 hpf), cellular proliferation is predominant, while the gastrula stage (5–24 hpf) is characterized by significant gene expression changes and morphological development. For instance, increased cell numbers during the blastula stage could influence the extent of teratogenic outcomes observed later in the gastrula stage. Factors such as dosage, duration of exposure, and environmental context are also critical modulators of these effects, highlighting the importance of understanding VPA’s impact on specific developmental stages.

Epigenetic changes reported for a wide variety of genes involved in gene regulation were also associated with exposure to VPA. These changes suggest that the formation of the CNS and the cardiovascular system are compromised at the genetic level, and these changes translate into alterations in their form and function. For example, the reduction in heart rate dependent on the increase in dose is consistent with a model of cardiac dysfunction, such as alterations in heart rate, structure, edema, decreased heart rate, and biomarkers of cardiac dysfunction [59]. The cardiovascular system in zebrafish begins its formation at approximately 5 hpf and ends at approximately 48 hpf, so VPA could have different effects in terms of its severity during different critical moments in the development of the heart [60,61]. However, our analysis did not detect any significant relationship between the period of exposure to VPA and cardiac function, which could suggest a wider window of vulnerability to external insults in the formation of this system, although underlying methodological differences with respect to the assessment that may not be sensitive to the period of exposure are not ruled out. For this reason, it would be worthwhile to explore in depth the susceptibility of the cardiac system to key moments of exposure to disruptors.

In a similar way, the manifestations of the behavioral domain reported individually were not significant in our meta-analysis. This could imply significant methodological differences that would not allow a joint statistical comparison. Although individual social deficits are commonly reported in this model. Standardized protocols are necessary to allow a joint comparison of the results with the objective of allowing accurate explanations.

The changes induced by VPA at the neuroanatomical level in different structures are accompanied by alterations in neuronal function and communication systems, an aspect widely described in other models of neurodevelopmental disorders [62]. These compromises in neural function may explain the previously reported behavioral deficits. Specifically, the inhibitory/excitatory balance has been proposed as a possible biomarker of ASD [6,63]. In this sense, the integral functional and orchestrated participation of different neurotransmission systems is crucial for proper brain function. Therefore, alterations in any of these systems suggest possible pharmacological targets for the treatment of these disorders.

The changes in the regulation of neurotransmitters discussed here are congruent with the changes in the expression of receptors and protein systems necessary for neuronal function. Specifically, changes in the *SHANK* system have also been strongly related to animal models of ASD and evidence from human studies [64,65]. The participation of this protein complex in neurotransmission, inhibitory/excitatory balance, neuroplasticity, and social behavior has focused attention on the pathophysiology that causes the behavioral manifestations of ASD and other neurodevelopmental disorders.

On the other hand, changes in the expression of *asdl* and *mbd5* did not reach statistical significance in our meta-analysis. However, the results of different experiments reported significant changes in the expression of these genes related to exposure to VPA individually, which could be due to methodological variability, or the small number of trials considered. However, there are other reports that individually revealed significant changes in the gene regulatory system, which supports the idea of epigenetic changes [22,44,45]. In the case of *asdl*, which actively participates in the purinergic system, individual assays report changes in the expression of this gene, and related nucleosides in this system, which does not rule out the effect of VPA on the regulation of this system.

According to the present analysis, exposure to VPA clearly induces generalized alterations in early development when it is administered during critical periods of the nervous system. In this sense, changes in cell proliferation, migration, and differentiation processes could be compromised by exposure to VPA due to its action on the HDAC epigenetic regulation system. These changes in processes crucial for the formation of the organism could explain macrostructural abnormalities at the level of the bone, cardiovascular, and cerebral systems. This, in turn, could partially explain the altered functionality at different levels, such as heart rate, neuronal communication, and neurotransmitter expression. Alterations in neuronal communication and its functionality have been proposed as underlying mechanisms of behavioral dysfunctions in the social and cognitive domains.

## 5. Conclusions

In conclusion, the present meta-analysis provides evidence of the teratogenic effects of VPA in zebrafish. In this sense, the high methodological heterogeneity between studies is highlighted, which emphasizes the need to standardize experimental protocols to improve the possibility of making comparisons between the data and results obtained. However, despite this variability, common effects of VPA in early development, such as alterations in heart rhythm, neurological changes, and epigenetic regulation, have been identified. These findings suggest that exposure to VPA during critical periods of development may compromise essential processes such as cell proliferation, migration, and differentiation, contributing to macrostructural and functional abnormalities. Understanding these mechanisms is crucial for the development of preventive and therapeutic strategies for the management of neurodevelopmental disorders associated with exposure to VPA. Future research should focus on standardizing methodologies that adapt and reduce the adverse effects of VPA in early development.

## Data Availability

The datasets used or analyzed during the current study are available from the corresponding author upon reasonable request.
